# Psychometric properties of the EDINA questionnaire for assessing self-esteem in childhood in a Peruvian population

**DOI:** 10.3389/fpsyg.2025.1661570

**Published:** 2026-03-12

**Authors:** Neftali Torres-Tapia, Calixto Tapullima-Mori, Miliam Quispe-Vargas, Eddy Wildmar Aquize Anco

**Affiliations:** 1 EP. Psicología, Facultad de Ciencias de la Salud, Universidad Peruana Unión, Juliaca, Peru; 2 Escuela de Psicología, Universidad Peruana Unión, Tarapoto, Peru; 3 Escuela Profesional de Educación de Idioma Extranjero, Universidad Nacional Jorge Basadre Grohmann, Tacna, Peru

**Keywords:** children, instruments, Peru, questionnaire, self-concept, self-esteem, self-image, validation

## Abstract

This research aimed to establish and validate the psychometric properties of the EDINA Questionnaire in a Peruvian sample, given the impact of healthy self-esteem on emotional development. A sample of 630 children, aged between 4 and 7 years, selected from educational institutions in the Puno region, participated. Content validity was confirmed using Aiken’s V (V ≥ 0.80), assessed by a panel of 10 experts who evaluated criteria such as clarity, congruence, and cultural appropriateness. Three items were adjusted after analyzing the results of the 95% confidence interval. Exploratory factor analysis (EFA) resulted in a unidimensional factor solution in which all items presented loadings between 0.6 and 0.8; this procedure indicated a Kaiser–Meyer–Olkin value of KMO = 0.978 and a significant Bartlett’s test of sphericity (*p* = 0.000); therefore, there were sufficient inter-item correlations to support the use of this procedure. Additionally, the absence of multicollinearity was evidenced by correlation coefficients ranging between 0.4 and 0.7 in the correlation matrix. Regarding confirmatory factor analysis, the results indicate that the multifactorial model shows higher incremental fit indices (CFI = 0.95 and TLI = 0.947) than the unidimensional model (CFI = 0.948 and TLI = 0.937), indicating an adequate fit to the sample. Regarding the absolute fit index, the multifactorial model (RMSEA = 0.060) shows a better fit than the unidimensional model (RMSEA = 0.066); however, the unidimensional model is more acceptable.

## 1 Introduction

Currently, strengthening self-esteem during childhood is considered essential for integral development, as it impacts children’s ability to face challenges, build positive relationships, and achieve personal goals. In this regard, factors such as cultural norms, bullying, social anxiety, symptoms of anxiety and depression, and difficulties in emotional regulation contribute to low self-esteem (Lacroix et al., 2023).

According to a report by the United Nations (NU, 2024), at least one in seven children or adolescents between the ages of 10 and 19 suffers from mental disorders, including behavioral alterations, with one-third of these appearing before the age of 14. Similarly, the World Health Organization (WHO, 2024) states that 20% of children and adolescents worldwide have mental health problems, with nearly half showing symptoms before the age of 14. Suicide is the sixth leading cause of death among children aged 5 to 14, manifesting through intense feelings of stress, confusion, self-doubt, pressure, and other expressions. Additionally, the isolation experienced during the pandemic has interfered with young people’s ability to relate to others—an aspect crucial for strengthening their identity, building self-esteem, and social adaptation (Cortés-Cortés, 2022). In this sense, considering that childhood is a key stage for the development of adequate self-esteem, it influences how a person interacts or performs in various areas such as family, social, academic, among others.

In the Peruvian context, the Ministry of Health (MINSA, 2023) reported having treated more than 23,600 cases of behavioral disorders in children and adolescents. In 2022, it also conducted an assessment using the PSC-17 scale to measure emotional, behavioral, and attention problems in children. The results showed that 3 out of 10 children aged between 1.5 and 5 years (32.2%) presented some type of mental health problem, placing them in the risk category. Similarly, in the group aged 6 to 11 years, 32.5% of children were also at risk of presenting at least one of these difficulties. Studies and reports on children’s self-esteem are generally scarce in our context; however, the work carried out by mental health professionals considers that, within the educational context, learning difficulties and school dropout are factors associated with low self-esteem, as they are determinant in academic performance (Acun, 2020).

Regarding the measurement of self-esteem, the literature presents studies that have examined the psychometric properties of instruments for evaluating self-esteem in adults and adolescents; however, there is limited evaluation in child populations (López-García et al., 2020). In the United Kingdom, Wood et al. (2021) conducted a study aiming to measure the internal consistency of the Rosenberg Scale, using a sample of 711 children (mean age = 9 years) and a subsample of 417 who also completed a life satisfaction scale. A confirmatory factor analysis (CFA) and tests of factorial invariance by gender were carried out. The findings indicated an adequate fit for the global self-esteem model (*χ*^2^ = 77.22; CFI = 0.961; RMSEA = 0.051) and acceptable internal consistency (*α* = 0.79). No significant gender-related differences in self-esteem were found, although smaller differences were detected in children aged 9 to 12 compared to those aged 7 to 8. Additionally, self-esteem showed a positive and meaningful relationship with life satisfaction. This research indicates that the CRSES version is a reliable tool to assess self-esteem in the context of child well-being.

In Spain, the adaptation and psychometric characteristics of the Spanish version of the Child and Youth Resilience Measure (CYRM-32) were carried out in young people at risk of social exclusion. Using a mixed-methods approach, the research included translation, cultural adaptation, and psychometric evaluation of the scale in three stages. The results of the confirmatory factor analysis (CFA) and exploratory factor analysis (EFA) indicated a three-factor structure, which explained 30.8% of the total variance: “Family Interaction,” “Interaction with Others,” and “Individual Skills.” Likewise, the scale showed appropriate internal consistency, with a total alpha of 0.877. In addition, a significant and positive correlation was found between resilience and self-concept, particularly in the social, academic, and family dimensions, suggesting that higher self-esteem contributes to greater resilience in youth. In conclusion, the study highlights the importance of cultural context in resilience processes and validates the CYRM-32 scale as a reliable tool for measuring resilience in vulnerable populations in Spain.

Lagonell et al. (2018) developed and tested a self-esteem scale intended for students in both Catalonia and Venezuela. Data collection took place in two stages: first, a pilot test with 300 students, followed by a second phase with 636 participants aged 6 to 9 years. Through factor analysis, the final instrument consisted of 19 items grouped into four components: social acceptance, negative feelings, school satisfaction, and self-worth. Additionally, a factorial verification was conducted in the Venezuelan sample, where the maximum likelihood estimation method indicated a good fit (CFI = 0.94; RMSEA = 0.039). The results of this scale showed variations in self-esteem aspects at the beginning of the educational stage, comparing two different situations in two different contexts. However, important aspects such as family, physical, and personal self-esteem were not considered (Juul, 2003).

In Argentina, Molina et al. (2011) conducted a study emphasizing the importance of having instruments that allow the evaluation of self-perceptions and self-esteem. The procedure involved adapting the Self-Perception Profile for Children (Harter, 1985) for use with children in Buenos Aires (C. A. B. A.). In the initial phase, both linguistic and conceptual equivalence were achieved. In the second stage, the local version of the scale was applied to a group of 219 primary school children aged 8 to 12 from private institutions in C. A. B. A., of both sexes, with a mean age of 10.34 years (SD = 1.77). The ability to differentiate components, reliability, and structural and content validity were assessed. The findings indicated that the instrument is valid and reliable, facilitating the assessment of self-perceptions. The SPPC evaluates the child’s self-image across various domains, as well as general self-esteem.

A comprehensive and specific approach to the study of self-esteem assessment in children is offered by Serrano (2014), the author of the EDINA questionnaire. The purpose of this study was to create and validate an instrument to assess self-esteem in children aged 3 to 7 years, evaluating its validity in terms of content, comprehension, and construct. The first analysis was carried out with the participation of nine experts using the Delphi method, followed by a pilot study with 250 children, which allowed for adjustments to the experimental version. The final version consisted of 21 items distributed across five subscales (physical, personal, social, academic, and family) and was applied to a group of 1,757 children. The findings showed excellent internal consistency (*α* = 0.803) and positive discrimination capacity of the items. Additional analyses indicated that self-esteem fluctuates depending on age, gender, socioeconomic status, and academic achievement, which are considered relevant determining factors. The instrument was also validated in 241 schoolchildren aged 3 to 7. Content validation was carried out through expert consensus using the Delphi method, while comprehension validity was determined through questionnaire application. The initial data collected via the Delphi method led to certain modifications suggested by expert judges. The questionnaire was refined to 18 items, which demonstrated appropriate modifications in terms of content and comprehension validity. The factorial study conducted revealed four dimensions of children’s self-esteem: physical, academic, socio-emotional, and family (Mérida et al., 2015).

In Mexico, the testing and analysis of the self-esteem scale known as “IGA-2000” was conducted in a sample of 767 grocery store employees from various municipalities in Jalisco, including Guadalajara and Zapopan. Using a descriptive and cross-sectional design, the scale showed an explained variance of 56.7% and a Cronbach’s alpha of 0.892, indicating high reliability. In the principal components analysis with Varimax rotation, the items clustered into a single factor, except for two outliers (items 10 and 16). This study supports the use of the scale as a unifactorial tool. The scale verification facilitates the acquisition of reliable information about its utility and interpretation, ensuring its appropriate application in similar contexts. In the same context, the Coopersmith Self-Esteem Inventory was adapted for Mexican elementary school students to measure self-esteem in children and adolescents. This process was guided by expert judgment, and reliability was assessed using Cronbach’s alpha, yielding an index of 0.813, indicating an appropriate level of utility to measure self-esteem across four dimensions: general, social, academic, and home.

Self-concept is a variable that has been considered in order to guide the assessment of self-esteem. For this reason, López-García et al. (2020) examined the psychometric characteristics of the adapted version of the Piers-Harris 2 Self-Concept Scale in a selected sample of 493 students aged 7 to 12, of whom 50.5% were female, with an average academic level of fourth grade. Internal consistency was calculated using the KR-20 formula, obtaining an index of 0.87, similar to previous studies. Additionally, the analysis based on the Rasch model showed that 58 items on the scale demonstrated notable characteristics of validity, reliability, and adaptability. These findings support the application of the scale in Mexican groups of children aged 7 to 12, demonstrating that it possesses appropriate psychometric properties for this population.

In Peru, there is a lack of research validating instruments for measuring self-esteem in children. According to our sociocultural context, the instruments and scales implemented in the Spanish context to assess self-esteem have not been fully adapted, making it difficult to access complete versions. Most questionnaires or scales are lengthy and contain vocabulary that is not easy for most children—especially those aged 3 to 7—to understand (Mérida et al., 2015). It is essential to develop appropriate tools to assess children’s self-esteem, thereby promoting their emotional and social well-being in a challenging context and providing a better understanding of individual behavior (Acevedo and Carrillo, 2010).

In a group of university students from Tarapoto, Carranza and Bermúdez-Jaimes (2017) studied the psychometric characteristics of the AF5 Self-Concept Scale, developed by García and Musitu, with the participation of 861 students. The Likert-type scale consists of 30 items divided into five aspects: academic, social, emotional, family, and physical self-concept. Initially, an exploratory factor analysis (EFA) with Varimax rotation was conducted, in which five factors accounted for 51.98% of the total variance. Subsequently, a confirmatory factor analysis (CFA) was carried out using structural equation modeling, including four specifications. The final results showed appropriate fit indices (RMSEA = 0.05; *p* = 0.05; TLI = 0.90; CFI = 0.92; CMIN/DF = 3.521; GFI = 0.92; and AGFI = 0.90). The instruments showed reliability levels ranging from 0.771 to 0.835. These findings are consistent with the initial results, thus confirming the AF5 as a valid and reliable instrument. These dimensions are closely related to the assessment of self-concept, as they address important aspects of self-esteem development and are similar to the dimensions of the EDINA questionnaire (Mérida et al., 2015).

In Cajamarca, a study was conducted by Sánchez-Villena et al. (2021) with the aim of analyzing the internal structure and reliability level of the Rosenberg Self-Esteem Scale (RSE). The scale was applied to 715 adolescents (51.3% female, *M* = 12.20). Six dimensions were identified through confirmatory factor analysis, aligning with factorial structures reported in previous research. The findings indicated greater adaptability of the unidimensional model, controlling for the impact of reverse-worded items and eliminating item 8 due to its plausibility. Reliability was confirmed using the omega coefficient. The unifactorial structure remains a topic of discussion, but it was concluded that the instrument is valid and adequate for use.

Currently, self-esteem is defined as a vital factor in confidence, security, and personal value; thus, having positive self-esteem contributes to better interaction, perception, and tolerance. Likewise, it can be referred to as an individual’s positive or negative view of their entire being (Bagley and Mallick, 2001). Similarly, self-esteem refers to a person’s evaluation of themselves and corresponds to an evaluative or affective dimension, resulting from the combination of objective information about oneself and its subjective assessment (Serrano, 2014).

According to the review of existing scales, it is evident that methodological aspects such as age have not been adequately considered. Many of the questions and responses in these instruments require participants to be able to read and write independently in most situations. Self-esteem is fundamental to individual development; for this reason, some authors argue that, in addition to the family, school is a key environment where children develop both physically and psychologically. Self-esteem begins to form from internal and external personal experiences (Serrano et al., 2016).

The development of self-esteem results from the child’s meaningful experiences within their environment, including the family, educational, and social settings. Additionally, having a more comprehensive understanding of self-esteem at an early age allows for the timely correction of various disorders that could affect future development. Positive self-esteem in children also acts as a protective factor against the onset of mental health problems, as those with higher life satisfaction tend to show higher levels of self-esteem (Muñoz-Albarracín et al., 2023). Conversely, children prone to high levels of anxiety and depression tend to have lower self-esteem and difficulties with emotional regulation (Öz and Kıvrak, 2023).

Given the issues described above, there is a need to validate an instrument capable of assessing self-esteem in Peruvian children aged 4 to 7, as such work has not yet been thoroughly conducted. This study also contributes to the validation of a tool that could support the prevention and intervention of self-esteem issues in educational settings, helping to identify students’ self-esteem levels (Mérida et al., 2015).

The findings will support the development of integrated interventions to help children with mental health problems build confidence and belief in themselves during the process of personal identity formation (Lee and Lee, 2024). The literature identifies a significant issue: the lack of validated instruments for assessing self-esteem in children. This limits the accurate identification of self-esteem levels and the implementation of effective interventions. In other words, there is limited research on the psychometric properties of instruments designed to evaluate children’s developing strengths (Navarro-Pérez et al., 2023). Therefore, a valid tool is needed to measure children’s self-esteem so that the results can be used to help prevent childhood psychopathology (Weeland et al., 2019).

Recognizing the need for a validated instrument to assess self-esteem in Peruvian children, this study proposes the replication of the EDINA questionnaire, along with the validation of its factorial structure to ensure better comprehension of the items (Mérida et al., 2015). Although instruments exist for assessing self-esteem in adult populations, studies involving children are significantly fewer. One of the main reasons why children are often excluded from such assessments is the difficulty they face in understanding the items due to their length and the reading and writing skills required (Mérida et al., 2015). Moreover, studies conducted with child populations emphasize the importance of having contextually appropriate validated psychometric instruments, which facilitate a better understanding of children’s behavior (Acevedo and Carrillo, 2010). Empirical evidence also shows that among emotional-cognitive variables, self-esteem contributes significantly to academic success. Students with high self-esteem levels tend to perform better in reasoning, executive functioning, and motivation (Pascual et al., 2019). Students with higher self-esteem scores also achieve higher levels of cognitive and motivational functioning (Quílez-Robres et al., 2021).

## 2 Methodology

### 2.1 Study design and type

This is an instrumental study with an applied psychometric design, aimed at evaluating the validity and reliability of the tool used to examine the psychometric properties of the EDINA questionnaire (Ato et al., 2013).

### 2.2 Participants

The sample size was estimated based on the predictive power of the CFI, with a statistical power (1 - *β*) of 95%, an expected dropout rate of 10%, an average factor loading of 0.3, and an expected CFI of 0.95. The calculated minimum sample was 470; however, this threshold was exceeded due to the accessibility of the study population. A total of 630 children aged 4 to 7 years (323 girls and 307 boys) from educational institutions in the Puno Region were surveyed. Participants were selected using non-probabilistic convenience sampling (Hernández-Sampieri and Mendoza, 2019).

Informed consent was obtained from the children’s parents or legal guardians. Inclusion criteria were: children enrolled in an educational institution, aged between 4 and 7 years, of either sex, residing in the department of Puno, and with signed informed assent from parents or guardians. Exclusion criteria included: children under 4 or over 7 years of age; those whose parents did not sign the informed consent; and those living outside the Puno region. Children under 4 were excluded because, in the Peruvian context, early childhood education begins at the age of four.

### 2.3 Instrument

The EDINA Questionnaire for the Evaluation of Self-Esteem in Childhood, authored by Serrano et al. (2013) and Mérida et al. (2015), is designed to assess self-esteem in the physical, personal, academic, social, family, and overall domains among children aged 3 to 7 years (corresponding to the second cycle of early childhood education and the first cycle of primary education).

Its application takes approximately 10 min. It is administered individually with the help of an evaluator for children aged 3 and 4, and in small groups of five to six students for those aged 5, 6, and 7. The items use a 3-point Likert scale: No = 1 point, Sometimes = 2 points, and Yes = 3 points. The questionnaire provides both a global self-esteem score and scores for five specific dimensions: physical, social, personal, academic, and family. The original version demonstrates an adequate factorial structure (RMSEA = 0.047, GFI = 0.951; AGFI = 0.937) and a Cronbach’s alpha of 0.803.

### 2.4 Data collection process

Initially, authorization was requested from the original authors of the questionnaire (Mérida et al., 2015), who granted permission to carry out the validation process with a Peruvian sample. Subsequently, data collection took place between April and September 2024. The data were collected in person, coordinated through the principals of the participating educational institutions. After obtaining informed consent from parents, the instrument was administered by grade level, with each session lasting between 10 and 15 min.

### 2.5 Ethical considerations

This study was reviewed and approved by the university’s ethics committee under document number 2024-CE EPG-00050. Authorization was then obtained from each early childhood and primary educational institution (up to second grade), with the research project profile attached. This document outlined the study objectives and clarified the requirement for informed consent from parents, as the participants were minors. Additionally, informed assent was obtained from each child prior to administering the instruments.

### 2.6 Data analysis

The research was divided into three phases. In the first phase, content validity was established through expert judgment to assess whether the items corresponded to the proposed factors. In the second phase, a confirmatory factor analysis (CFA) was performed, evaluating various fit indices, such as the Comparative Fit Index (CFI), the Tucker-Lewis Index (TLI), as well as the RMSEA and SRMR with the ML estimator. An exploratory factor analysis was also conducted. Finally, construct reliability was determined using Cronbach’s alpha coefficient and its corresponding confidence intervals.

## 3 Results

### 3.1 Content validity

Content validity was assessed through the judgment of 10 expert psychologists who work with children, using Aiken’s V coefficient. Experts were provided with a validation form for the items, based on four criteria: clarity, coherence, contextual relevance, and construct control. Responses were recorded on a 7-point scale ranging from 0 to 6, where 0 = the validation criterion is not met, and 6 = the criterion is fully met. The responses were analyzed using Aiken’s V coefficient with 95% confidence intervals, with a lower bound of V ≥ 0.80 considered acceptable (Table 1).

**Table 1:** Descriptive analysis.

Item	Mean	S. D.	Sk	K	r item-test	Response rate		
						Yes	Sometimes	No
1	2.614	0.721	−1.539	0.686	0.668	75.5%	10.3%	14.1%
2	2.651	0.666	−1.665	1.281	0.742	75.8%	13.3%	10.7%
3	2.573	0.709	−1.347	0.303	0.719	70.1%	16.9%	12.8%
4	2.610	0.697	−1.496	0.694	0.632	73.3%	14.2%	12.3%
5	2.741	0.626	−2.196	3.164	0.811	84.1%	5.8%	10%
6	2.629	0.692	−1.581	0.930	0.754	75%	12.7%	12.2%
7	2.757	0.607	−2.303	3.684	0.804	84.9%	5.8%	9.2%
8	2.635	0.645	−1.544	1.065	0.804	72.6%	18%	9.2%
9	2.632	0.682	−1.585	0.991	0.718	74.7%	13.7%	11.6%
10	2.548	0.681	−1.201	0.118	0.611	65.6%	23.7%	10.8%
11	2.681	0.646	−1.812	1.803	0.802	78%	12%	10%
12	2.579	0.695	−1.356	0.386	0.646	69.8%	12.2%	12%
13	2.698	0.666	−1.939	2.072	0.807	81.4%	7%	11.6%
14	2.577	0.722	−1.378	0.312	0.685	71.4%	14.6%	13.8%
15	2.622	0.686	−1.543	0.864	0.761	74%	14.2%	11.8%
16	2.721	0.619	−2.036	2.673	0.820	81.1%	9.8%	9%
17	2.576	0.729	−1.380	0.289	0.699	71.9%	13.8%	14.3%
18	2.690	0.668	−1.893	1.920	0.772	80.6%	7.8%	11.6%
19	2.640	0.703	−1.646	1.040	0.731	77.1%	9.7%	13.1%
20	2.665	0.651	−1.723	1.524	0.786	76.5%	13.4%	10%
21	2.694	0.650	−1.895	2.031	0.786	79.8%	9.7%	10.5%

Based on these criteria, three items required modification: Item 7 due to clarity [V = 0.86; 95% CI (0.76; 0.96)], Item 11 for clarity [V = 0.86; 95% CI (0.76; 0.96)] and coherence [V = 0.88; 95% CI (0.78; 0.94)], and Item 17 for clarity [V = 0.88; 95% CI (0.78; 0.94)].

The following changes were made according to the experts’ suggestions:

- Item 7: “Me gusta dar muchos besitos” was modified to “Me gusta dar muchos abrazos a papá y mamá” (“I like giving lots of hugs to mom and dad”).
- Item 11: “Siempre entiendo lo que el maestro o la maestra me piden que haga” was changed to “Entiendo lo que mi profesora o profesor me enseñan” (“I understand what my teacher teaches me”).
- Item 17: “Me río mucho” was modified to “Me río mucho cuando estoy con mi familia o con mis amigos” (“I laugh a lot when I’m with my family or friends”).

### 3.2 Descriptive analysis

Table 1 describes the behavior of the items using measures of central tendency, dispersion, and distribution. The findings showed that the mean across all items was similar, ranging between 2.5 and 2.7. The highest standard deviations were found in Items 19, 17, 14, and 1. Skewness (Sk) and kurtosis (K) values revealed that 61.9% of the items did not meet the assumption of normality. Specifically, Items 1, 2, 5, 6, 7, 8, 9, 11, 13, 15, 16, and 18 violated the normal distribution assumption. Item-test correlations indicated moderate relationships ranging from 0.6 to 0.8. Regarding response frequency, between 65 and 80% of the participants selected responses such as “Yes” or “Sometimes” in relation to the indicators of self-esteem (Table 1).

## 4 Construct validity

### 4.1 Exploratory factor analysis

Mardia’s multivariate normality test, used to verify compliance with the EFA assumptions, showed a significance level below 0.05 for the KS statistic, justifying the use of the principal axis factoring method (Pérez and Medrano, 2010) due to the violation of the normality assumption. In addition, the “oblimin” rotation method was used, as it is one of the oblique methods appropriate when factors are interrelated (Table 2).

**Table 2:** Exploratory factor solution.

Item	Factor 1	Uniqueness
I16	0.838	0.298
I5	0.828	0.314
I7	0.823	0.322
I13	0.823	0.323
I11	0.819	0.330
I8	0.819	0.330
I20	0.803	0.356
I21	0.802	0.356
I18	0.788	0.379
I15	0.774	0.400
I6	0.769	0.409
I2	0.758	0.426
I19	0.747	0.442
I3	0.733	0.463
I9	0.727	0.471
I17	0.711	0.495
I14	0.695	0.517
I1	0.680	0.537
I12	0.658	0.566
I4	0.642	0.588
I10	0.620	0.616

These procedures yielded a one-dimensional factorial solution, where all items showed loadings between 0.6 and 0.8. The KMO value and Bartlett’s test results were 0.978 and 0.000, respectively, indicating sufficient inter-item relationships to proceed with the analysis.

Furthermore, the absence of multicollinearity was evidenced by correlation coefficients ranging from 0.4 to 0.7 in the correlation matrix.

### 4.2 Confirmatory factor analysis

Two factorial models of the test were evaluated. The first was a multidimensional model based on the five-factor structure identified in the original work by Mérida et al. (2015). The second was a unidimensional model suggested by the exploratory factor analysis.

The results showed that the multifactorial model exhibited better incremental fit indices (CFI = 0.95 and TLI = 0.947) than the unidimensional model (CFI = 0.948 and TLI = 0.937), indicating that the multifactorial model fits the sample well.

Regarding the absolute fit index, the multifactorial model (RMSEA = 0.060) demonstrated a better fit than the unidimensional model (RMSEA = 0.066) (Table 3).

**Table 3:** Fit indices of the original model.

Fit indices	Acceptable thresholds	Multifactorial model	Unidimensional model
*X* ^2^	–	583.8	700.8
df	–	179	189
*X*^2^/df	5	3.262	3.7
NFI	0.90	0.942	0.931
RFI	0.90	0.926	0.916
IFI	0.90	0.959	0.949
TLI	0.90	0.947	0.937
CFI	0.90	0.959	0.948
RMSEA	0.08	0.060	0.066
RMSEA (90% CI)	0.08	0.055–0.065	0.060–0.071

**Figure 1:**
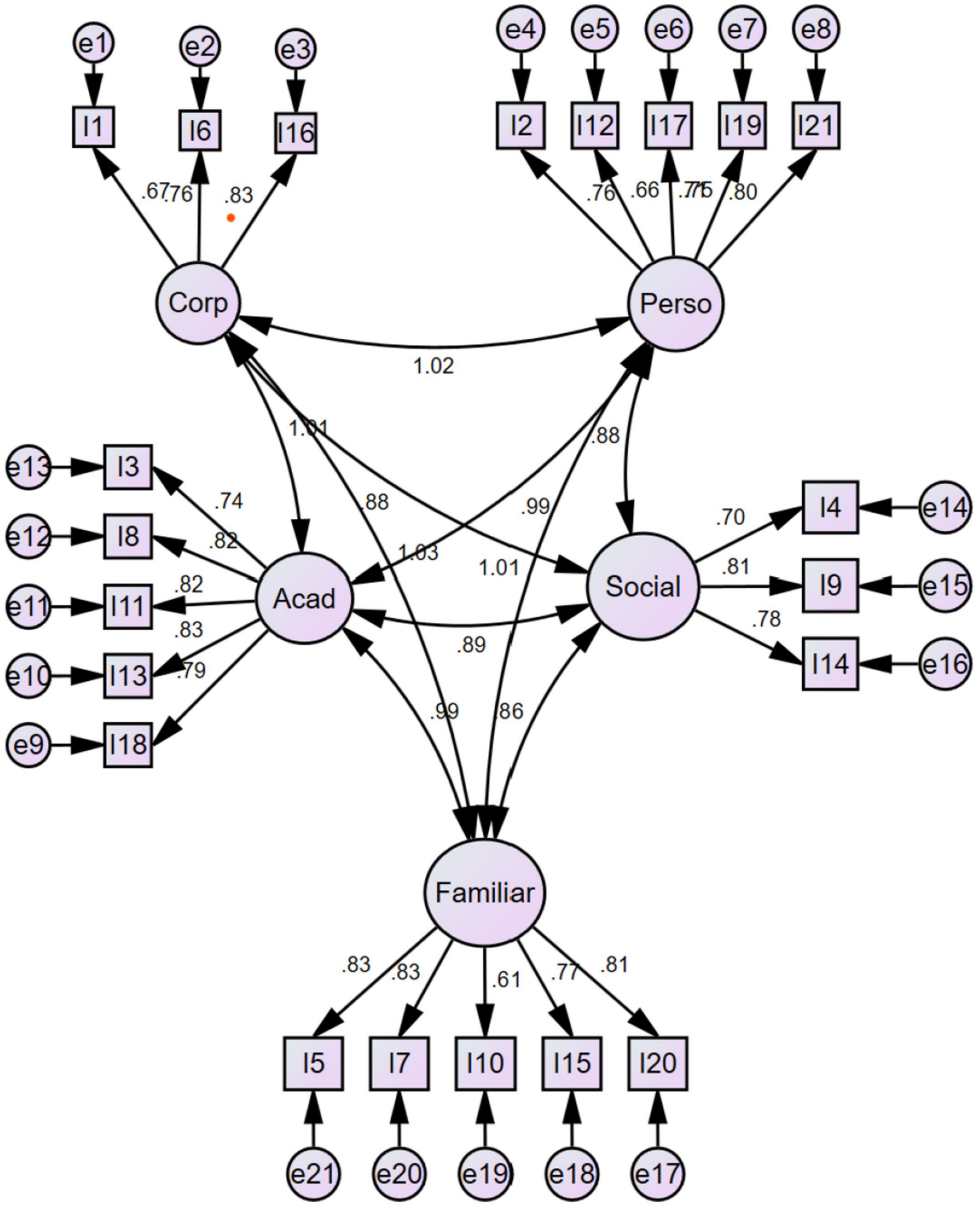
Flow diagram of the adjusted model.

Figure 1 shows the estimators of the multidimensional model, which demonstrated better fit indices. The findings confirmed that the items present estimators above 0.60, indicating their appropriate loading within each respective factor. Multicollinearity between factors was observed, particularly between the following dimensions:

- Body and personal self-esteem (1.02)
- Body and academic self-esteem (1.01)
- Academic and personal self-esteem (1.03)
- Family and personal self-esteem (0.99)

No residuals were covaried.

**Figure 2:**
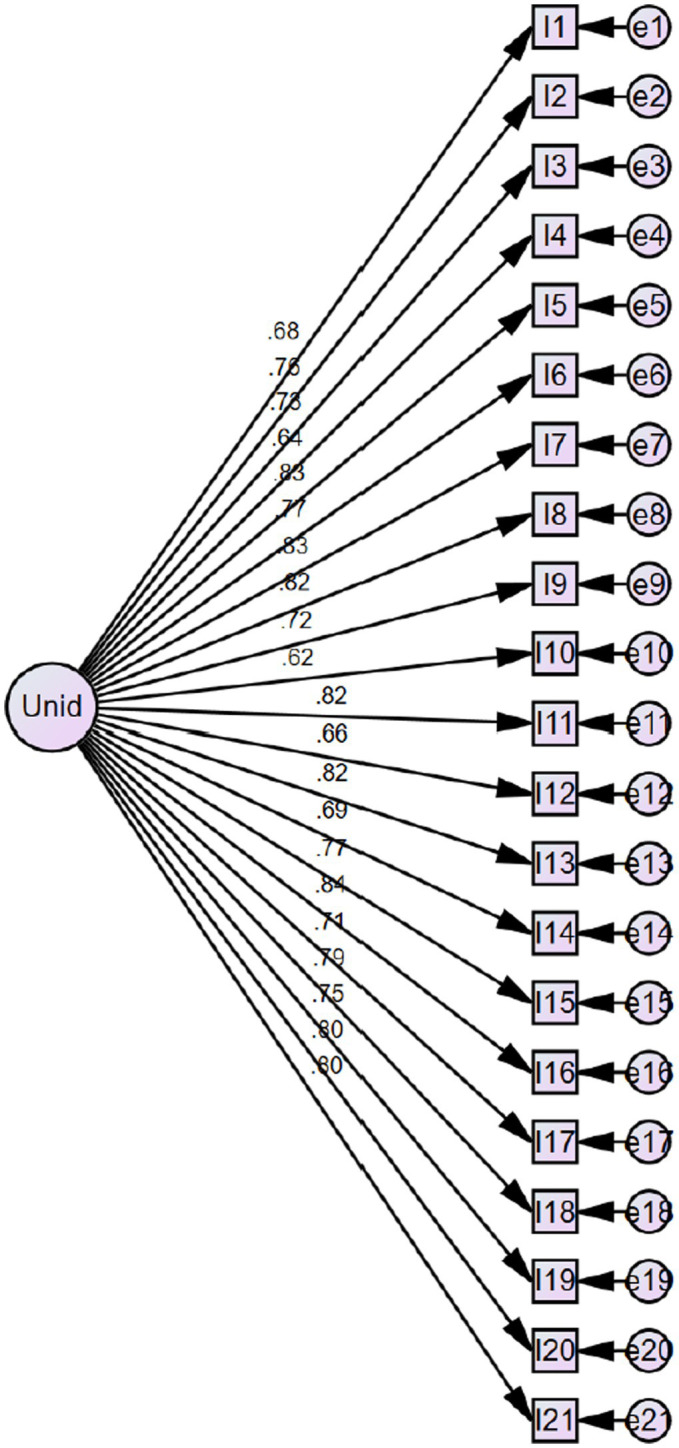
Flow diagram of the unidimensional model.

Given the possible existence of a single latent variable as suggested by the EFA, and the observed multicollinearity between factors, a unidimensional model was tested using CFA. Figure 2 shows the structural quality of this model, with all items presenting factor loadings above 0.60 and no residual covariances.

### 4.3 Easurement invariance via multigroup CFA

The differences observed in CFI, TLI, SRMR, and RMSEA did not exceed 0.01, indicating strong measurement invariance of the scale by gender in the unidimensional model (Table 4).

**Table 4:** Measurement invariance by gender through multigroup CFA (307 boys – 323 girls).

Levels	CFI	TLI	RMSEA	SRMR	ΔCFI	ΔTLI	ΔRMSEA
Configural	0.992	0.991	0.039	0.037			
Metric	0.994	0.993	0.034	0.046	0.002	0.002	0.005
Scalar	0.993	0.992	0.036	0.037	0.001	0.001	0.002
Strict	0.993	0.992	0.036	0.037	0	0	0

### 4.4 Internal consistency reliability

The first dimension, body self-esteem, reached a Cronbach’s alpha and McDonald’s omega coefficient of 0.795, with 95% confidence intervals ranging from 0.765 to 0.821 and from 0.823 to 0.976, respectively. The second dimension, personal self-esteem, reached an omega coefficient of 0.853 with a 95% CI from 0.835 to 0.871, while its Cronbach’s alpha was 0.852 with a 95% CI from 0.833 to 0.870. The third dimension, academic self-esteem, had an omega of 0.898 with a 95% CI from 0.885 to 0.910, and a Cronbach’s alpha of 0.897 with a 95% CI from 0.884 to 0.910. The fourth dimension, social self-esteem, reached an omega coefficient of 0.808 with a 95% CI from 0.782 to 0.834, and a Cronbach’s alpha of 0.805 with a 95% CI from 0.777 to 0.830. Finally, the fifth dimension, family self-esteem, reached an omega coefficient of 0.876 with a 95% CI from 0.861 to 0.892, and a Cronbach’s alpha of 0.877 with a 95% CI from 0.861 to 0.892. Overall, the test reached a Cronbach’s alpha of 0.965 with a 95% CI from 0.960 to 0.968, and an omega coefficient of 0.965 with a 95% CI from 0.961 to 0.969.

## 5 Discussion

The present study aimed to establish and validate the psychometric properties of the EDINA Questionnaire in a Peruvian sample, which evaluates the self-assessment a person makes of themselves and corresponds to an evaluative or affective dimension. Self-esteem in childhood is a crucial construct within the context of emotional and psychological growth, as it significantly impacts mental health and the ability to cope with life. Its importance extends to being a key indicator of positive psychological outcomes in later stages of life, including academic performance. However, accurately assessing children’s self-esteem faces considerable challenges due to the limited availability of tools that are valid, reliable, and culturally sensitive. Inadequate tools can lead to misinterpretations that either underestimate or overestimate children’s emotional well-being. Therefore, validating and developing specific instruments to assess self-esteem in this population is essential, as they offer a solid foundation for effective intervention.

To confirm internal consistency in the exploratory phase (EFA), a CFA was conducted using a factorial solution based on a total of 630 children. The structure that emerged from the EFA is justified according to the guidelines of Kyriazos (2018) when validating scales in a different population. The Mardia multivariate normality test, used to verify the assumption for EFA, showed a significance level below 0.05 for KS, which supports the use of principal axis factoring (Pérez and Medrano, 2010).

Regarding item analysis, skewness and kurtosis values were within expected limits, indicating that all items demonstrated excellent discrimination capacity. The exploratory factor analysis showed a good measure of sampling adequacy with KMO = 0.978 and Bartlett’s test *p* = 0.000 (Watkins, 2018). Furthermore, two factorial models of the test were examined: one multidimensional, considering the five factors proposed by Mérida et al. (2015), and one unidimensional.

This study validated the adapted version of the EDINA questionnaire in a Peruvian child sample, replicating and confirming the factorial structure and reliability observed in the original version by Mérida et al. (2015), who developed the instrument to measure self-esteem in children aged 3 to 7 in Spain, obtaining appropriate goodness-of-fit indices and internal consistency (RMSEA = 0.047, GFI = 0.951). Similarly, the study by Wood et al. (2021) in the United Kingdom using the Rosenberg scale showed good internal consistency and structural fit in a child population. In the same line, the results of the present study confirmed the multifactorial structure and high reliability of the EDINA questionnaire in the Peruvian context, with Cronbach’s alpha coefficients above 0.80, ensuring its accuracy and applicability (Serrano et al., 2013). However, the unidimensional model is the most appropriate.

Moreover, both exploratory and confirmatory factor analyses showed that the multifactorial model—including the dimensions of physical, personal, academic, social, and family self-esteem—provides a better fit than the unidimensional model. This finding aligns with international studies such as those by Lagonell et al. (2018) in Venezuela and Catalonia, and Molina et al. (2011) in Argentina, which emphasize the importance of multiple dimensions to capture the complexity of self-esteem in diverse educational contexts.

At the national level, the relevance of a validated and culturally adapted instrument is highlighted due to the lack of psychometric tools focused on Peruvian children. Therefore, studies that contribute to the understanding of positive body image are necessary (Hernández-Cruz et al., 2022), especially considering how educational and socioeconomic contexts influence the development of self-esteem, as shown by studies such as that of Carranza and Bermúdez-Jaimes (2017) in Peruvian adolescents.

One of the limitations of this study was the group administration of the questionnaire to children aged 4 to 7; however, the support of collaborators allowed for better guidance in completing the surveys. In conclusion, the validation of the EDINA questionnaire in Peru not only fills an important gap in the assessment of children’s self-esteem but also provides a reliable and culturally appropriate tool for diagnosis and intervention in educational settings, thus contributing to the development of evidence-based mental health policies for children.

## Data Availability

The raw data supporting the conclusions of this article will be made available by the authors, without undue reservation.
